# Study on the Adsorption of CuFe_2_O_4_-Loaded Corncob Biochar for Pb(II)

**DOI:** 10.3390/molecules25153456

**Published:** 2020-07-29

**Authors:** Tianci Zhao, Xiaolong Ma, Hao Cai, Zichuan Ma, Huifeng Liang

**Affiliations:** 1 College of Chemistry and Material Science, Hebei Normal University, Shijiazhuang 050024, China; zhaotianci152@163.com (T.Z.); hao_cai_515@163.com (H.C.); 2 School of Environmental Science and Engineering, Hebei University of Science and Technology, Shijiazhuang 050018, China; maxiaolong2410@163.com; 3 College of Chemistry and Chemical Engineering, Xingtai University, Xingtai 054001, China

**Keywords:** corncob biochar, CuFe_2_O_4_, load, adsorption, Pb(II)

## Abstract

A series of the magnetic CuFe_2_O_4_-loaded corncob biochar (CuFe_2_O_4_@CCBC) materials was obtained by combining the two-step impregnation of the corncob biochar with the pyrolysis of oxalate. CuFe_2_O_4_@CCBC and the pristine corncob biochar (CCBC) were characterized using XRD, SEM, VSM, BET, as well as pH_ZPC_ measurements. The results revealed that CuFe_2_O_4_ had a face-centered cubic crystalline phase and was homogeneously coated on the surface of CCBC. The as-prepared CuFe_2_O_4_@CCBC(5%) demonstrated a specific surface area of 74.98 m^2^·g^−1^, saturation magnetization of 5.75 emu·g^−1^ and pH_ZPC_ of 7.0. The adsorption dynamics and thermodynamic behavior of Pb(II) on CuFe_2_O_4_@CCBC and CCBC were investigated. The findings indicated that the pseudo-second kinetic and Langmuir equations suitably fitted the Pb(II) adsorption by CuFe_2_O_4_@CCBC or CCBC. At 30 °C and pH = 5.0, CuFe_2_O_4_@CCBC(5%) displayed an excellent performance in terms of the process rate and adsorption capacity towards Pb(II), for which the theoretical rate constant (k_2_) and maximum adsorption capacity (*q*_m_) were 7.68 × 10^−3^ g·mg^−1·^·min^−1^ and 132.10 mg·g^−1^ separately, which were obviously higher than those of CCBC (4.38 × 10^−3^ g·mg^−1^·min^−1^ and 15.66 mg·g^−1^). The thermodynamic analyses exhibited that the adsorption reaction of the materials was endothermic and entropy-driven. The XPS and FTIR results revealed that the removal mechanism could be mainly attributed to the replacement of Pb^2+^ for H^+^ in Fe/Cu–OH and –COOH to form the inner surface complexes. Overall, the magnetic CuFe_2_O_4_-loaded biochar presents a high potential for use as an eco-friendly adsorbent to eliminate the heavy metals from the wastewater streams.

## 1. Introduction

With the development of industry and technology, large quantities of heavy metals are getting widely used in metallurgical, electroplating, and battery industries, among others. Thus, significant amounts of heavy metals are inevitably released into wastewaters. However, the discharge of such wastewaters into the environment is strictly controlled by various laws considering their high toxicity and persistence [1,2]. As a result, effective methodologies and materials are needed to be developed for the effective elimination of heavy metal ions from contaminated water streams.

Conventional processes for the removal of heavy metal ions from wastewater streams involve ion exchange, precipitation reaction, reverse membrane osmosis, electrolytic deposition, adsorption, etc. Among them, adsorption is preferred for its inexpensive process, simplicity, and effectiveness in removing the heavy metals. Moreover, the adsorption processes can be utilized under various conditions [3,4]. Activated carbon, silica gels, chitosan, titania, iron oxide, hydroxyapatite, and manganese (IV) hydroxide are among the conventional absorbents [5,6,7,8,9]. However, these suffer from the shortcomings such as tendencies for oxidation and aggregation, limited adsorption capacity, low selectivity, and high cost [7]. Thus, the further development of highly efficient, abundant, and magnetically separable adsorbent materials has attracted much attention [10,11].

Biochar, a low-cost biomass-derived carbonaceous material, is an effective alternative to address the challenge of the water pollution of heavy metal [12]. In general, the biochars prepared directly from the biomass raw materials do not exhibit high adsorption capacity for capturing toxic metal ions. Thus, emphasis has further been focused on the pristine biochar modification [13]. Importantly, the preparation of the magnetic biochars has proved to be one of the most effective biochar modifications [14]. Use of the biochar-based magnetic materials to remove the heavy metal pollutants from the aqueous solutions not only exhibits high removal performance but also enables the spent adsorbents to be easily disposed using a low strength magnetic separation device [14]. In the recent literature, studies concerning the magnetic biochars used for controlling the heavy metal pollution, a variety of magnetic components were loaded/coated on the biochars derived from a wide range of biomass sources, thus, improving the structure and functionality [14,15,16,17,18,19,20,21]. The most commonly utilized magnetic substances are magnetite (Fe_3_O_4_) and maghemite (γ-Fe_2_O_3_). Son et al. reported the use of the mixed iron oxide particles (Fe_3_O_4_ and γ-Fe_2_O_3_) as magnetic components to prepare both kelp and hijikia magnetic biochars for the adsorption of copper, cadmium, and zinc ions [20,21]. A few research studies have reported the synthesis of manganese ferrite (MnFe_2_O_4_)/biochar composites via different techniques such as thermal decomposition, hydrothermal carbonation, sol-gel/pyrolysis, or co-precipitation for efficiently adsorbing Pb^2+^, Cu^2+^, Cd^2+^, and Sb^3+^ [22,23,24,25]. Similarly, cobalt ferrite (CoFe_2_O_4_) was combined by Reddy et al. with the pine bark biochar for achieving the magnetic biochar material with excellent adsorption performance of Pb(II) and Cd(II) [26]. Recently, Wang et al. reported the preparation of magnetic greigite/biochar composites (MGBs) using less-commonly used Fe_3_S_4_ nanosheets as a magnetic modifier, thereby, demonstrating the efficient removal of Cr (VI) on MGBs [17].

Based on the literature, it can be concluded that a limited variety of magnetic modifiers has been used in the fabrication of magnetic biochars. Therefore, exploration of the other types of magnetic modifiers for synthesizing magnetic biochars is needed in the future. In our previous study [25], a magnetic MnFeO_x_-loaded corncob biochar (MnFeO_x_@CCBC) was synthesized by combining the two-step impregnation of corncob biochar with the pyrolysis of oxalate, which demonstrated enhanced adsorption capacity. As a continuation of the last study, this study focuses on the use of spinel copper ferrite (CuFe_2_O_4_) for producing a novel magnetic biochar composite for enhanced removal ability of the heavy metal ions from the aqueous solutions. To date, CuFe_2_O_4_ and its derived composites have been extensively used for the abatement of various pollutants such as organic dyes [27,28,29,30,31], anions [31,32,33,34], arsenic [31,35,36], heavy metal ions, and organic toxicants [30,31,37,38], thus, implying that CuFe_2_O_4_ exhibits optimal properties for environmental applications. Therefore, introducing CuFe_2_O_4_ modifier into biochar may reach the expected requirements for adsorbents such as good magnetic separability, good adsorption capacity, eco-friendly character, and low budget.

The objective of this work was to study the feasibility of employing CuFe_2_O_4_ as a magnetic modifier for achieving the magnetically-separable CuFe_2_O_4_-loaded corncob biochar (CuFeO_x_@CCBC) for removing Pb(II) from the aqueous medium and to clarify its adsorption mechanism. The Pb(II) adsorption behavior on CuFeO_x_@CCBC was studied under various experimental conditions using batch method. The adsorption equations including the Langmuir and Freundlich isotherms as well as the typical kinetic models were used to analyze the experiment data. In addition, various analytical methods were implemented for the characterization of the as-fabricated samples and elucidation of the process mechanisms. Overall, this work provides a beneficial approach for the development of a new class of biochar-based adsorbent material.

## 2. Results and Analysis

### 2.1. Characterization of CuFe_2_O_4_@CCBC and Corncob Biochar (CCBC)

As can be seen from the Section 3.1, the synthesis of CuFe_2_O_4_ coating and modification of CCBC were achieved via a (Cu, Fe) binary oxalates precursor, which includes three sub-steps. First, CCBC support was impregnated with equal-volume ethanol-aqueous solution (80%, *v*/*v*) containing stoichiometric amount of copper and iron ions, resulting in a uniform distribution of copper and iron ions within the CCBC matrix (including surfaces and pores). Next, the binary oxalates were produced on the CCBC support through the oxalic acid solution impregnation. Finally, the binary oxalates were decomposed and converted to CuFe_2_O_4_ in the subsequent pyrolysis process, thus, achieving the CuFe_2_O_4_@CCBC composite. The SEM images of CCBC and CuFe_2_O_4_@CCBC(5%) are displayed in Figure S1a,b, respectively. As can be seen, the prepared materials were composed of irregular grains with obvious differences in size and morphology. The grain size was within the range of 5–25 μm for CCBC and 8–40 μm for CuFe_2_O_4_@CCBC(5%), which was consistent with the general characteristics of the biochar-based materials [13,14]. The particle size enlargement of the composite was observed after coating CuFe_2_O_4_ owing to the thermal decomposition of Fe/Cu oxalate precursors, thus, improving the surface and pore structure. Furthermore, as can be judged from the mapping diagrams of the Cu and Fe elements of the CuFe_2_O_4_@CCBC(5%) sample in Figure S1c,d, the CuFe_2_O_4_ modifier was uniformly coated onto the surface of CCBC, with no dominant aggregation, which led to an increase in the adsorption sites and an improvement in the adsorption performance.

**Figure 1:**
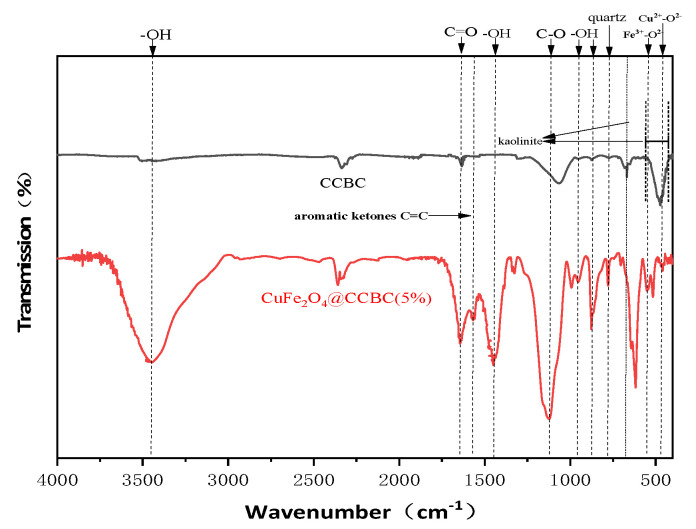
FTIR spectra of corncob biochar (CCBC) and CuFe_2_O_4_-loaded corncob biochar (CuFe_2_O_4_@CCBC)(5%).

FTIR measurement was carried out to confirm the production of CuFe_2_O_4_ and its effect on the surface functional groups (Figure 1). It was observed that the spectral peaks of CuFe_2_O_4_@CCBC(5%) had high intensity. The absorbance band at 3435 cm^−1^ is assigned to the –OH stretching vibration [15,25]. The absorbance peaks at 1640 and 1560 cm^−1^ are ascribed to the C=O vibrations of the carboxyl and aromatic ketones in the biochar frame [15,25]. The 1120 cm^−1^ peak is attributed to the C–O ether stretching vibration [15]. These results prove that the surface modification leads to abundant oxygen-containing groups on the interface of the biochar-based composite. In the spectrum of CuFe_2_O_4_@CCBC(5%), the absorbance bands at 570 and 460 cm^−1^ are vibrations of the Fe^3+^–O^2−^ and Cu^2+^–O^2−^ complexes, respectively [39,40], while the peaks at 870, 970, and 1457 cm^−1^ are the characteristic peaks of M–OH (M is Fe/Cu) due to bending vibration [40,41], indicating the formation of CuFe_2_O_4_ on the biochar-based composite. In the IR spectrum of CCBC, the strong peak at 450–515 cm^−1^ and weak peak at 670 cm^−1^ are attributed to the possible presence of kaolinite [42], and the possible presence of quartz may be inferred from the little peak at 782 cm^−1^ [42]. However, a stronger characteristic peak of quartz but no absorbance bands of kaolinite were observed in the infrared spectrum of CuFe_2_O_4_@CCBC(5%), implying that the concomitant disassociation and re-organization of the mineral fractions in the CCBC sample took place during the modification process.

The XRD patterns of CCBC and CuFe_2_O_4_@CCBC(5%) samples are shown in Figure S2. From the pattern of CCBC, quartz (with two moderately strong peaks at 2θ around 26.7° and 40.5°) was observed as the predominant crystalline phase [42,43], which agrees with the FTIR findings. No kaolinite and biochar peaks were detected, indicating their amorphous nature. In the XRD pattern of CuFe_2_O_4_@CCBC(5%), the 2θ values at 20.2°, 29.3°, 37.8°, 43.7°, 56.9°, and 64.0° could be indexed to the (101), (112), (202), (220), (321), and (224) crystal faces of a face-centered cubic CuFe_2_O_4_ (JCPDS No. 34-0425) [44], further demonstrating that CuFe_2_O_4_ was successfully covered on the surface of the biochar-based composite. Additionally, the peak of quartz was noted to shift from 26.7° (2θ) to 27.2° and was relatively stronger than that of CCBC, possibly due to the re-organization of its structure, which is also consistent with the FTIR characterization.

**Figure 2:**
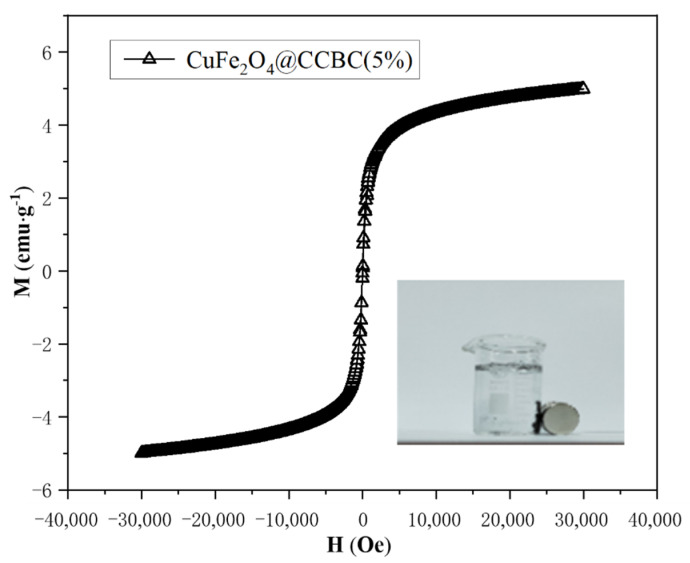
Hysteresis loop of CuFe_2_O_4_@CCBC(5%).

The formation of CuFe_2_O_4_ coating endows magnetism to CuFe_2_O_4_@CCBC(5%), which can be proved by its hysteresis loop (shown in Figure 2). The CuFe_2_O_4_@CCBC(5%) has a saturation magnetization (Ms) of 5.75 emu·g^−1^ and a coercive force (Hc) of 154.35 Oe. As expected, the magnetism intensity of the developed CuFe_2_O_4_@CCBC material is sufficient for its swift recovery from the treated solution by means of a small magnet, as shown in Figure 2. This demonstrates the potential of recycling of the magnetic biochar-based adsorbent in the wastewater remediation process.

The textural properties of the as-prepared biochar-based adsorbents were investigated by the N_2_ adsorption-desorption analysis at the boiling point of nitrogen. The nitrogen adsorption-desorption isotherms of CCBC and CuFe_2_O_4_@CCBC samples with different loading amounts (3, 5, and 8%) are schematically shown in Figure S3. The typical properties, including the specific surface area, total pore volume and average pore diameter, are presented in Table S1.

According to the IUPAC classification, the pristine CCBC sample demonstrates a typical type III isotherm; however, the modified CuFe_2_O_4_@CCBC composites exhibit type IV isotherms and style H3 hysteresis loops, spanning a broad relative pressure region and resembling the appearance of slit pores [40]. From Table 1, the CuFe_2_O_4_ coating and an increase in the loading amount are observed to result in an enhanced specific surface area (from 17.1 to 75.0 m^2^·g^−1^) and pore volume (from 0.039 to 0.082 cm^3^·g^−1^) as well as a reduced pore size (from 7.93 to 1.89 nm), which confirms that the CuFe_2_O_4_@CCBC composites have a mesoporous or microporous structure and contain a higher pore volume. In addition, it is observed that the surface area of 8% CuFe_2_O_4_@CCBC (75.0 m^2^·g^−1^) is lower than 5% CuFe_2_O_4_@CCBC (60.7 m^2^·g^−1^), which might be associated with the aggregation of the excess CuFe_2_O_4_ nanoparticles.

The ΔpH–pH_0_ curves of both CCBC and CuFe_2_O_4_@CCBC(5%) samples are shown in Figure S4. As can be seen, the pH_ZPC_ of CuFe_2_O_4_@CCBC(5%) (7.0) was 2.5 units lower than that of CCBC (9.5), reflecting that the number and strength of the protic groups (e.g., –OH and –COOH) in the CuFe_2_O_4_@CCBC(5%) structure is significantly increased, thus, leading to the sorption of the heavy metal ions through the ion exchange (with H^+^) or complexation mechanisms [45,46].

### 2.2. Effect of CuFe_2_O_4_ Loading Amount on Pb(II) Adsorption Capacity

**Figure 3:**
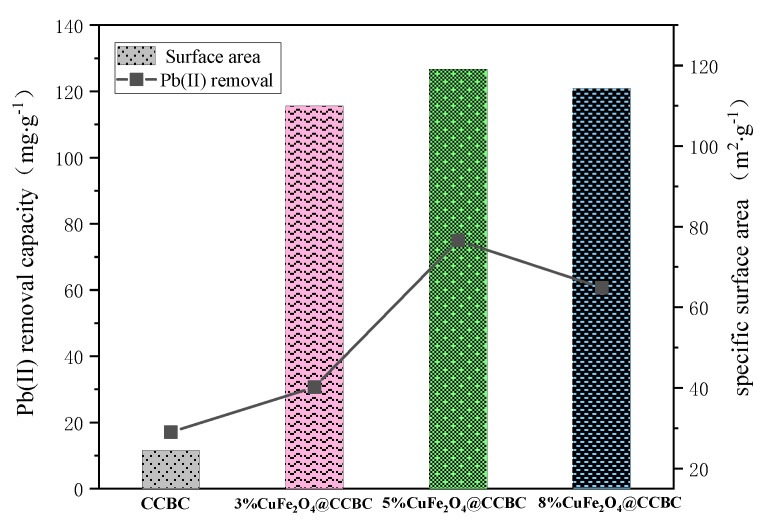
Comparison of the Pb(II) removal capacity and specific area of the samples.

In order to evaluate the improvement of the lead ion removal ability resulting from the CuFe_2_O_4_ coating, batch adsorption tests were carried out by adding 20 mg of the sample into 30 mL of the 500 mg·L^−1^ Pb(II) solution (pH = 5.0) at 30 °C. As shown in Figure 3, the CuFe_2_O_4_@CCBC composites showed significantly higher Pb(II) removal capacity than the pristine CCBC sample (from 11.50 mg·g^−1^ to 115.58, 126.67, and 120.90 mg·g^−1^), with CuFe_2_O_4_@CCBC(5%) exhibiting the best performance. A positive correlation between the Pb(II) removal capacity and specific surface area was observed among the tested samples. Overall, these results confirm the potential of CuFe_2_O_4_@CCBC(5%) as an effective adsorbent of heavy metal.

### 2.3. Adsorption Kinetic Analysis

**Figure 4:**
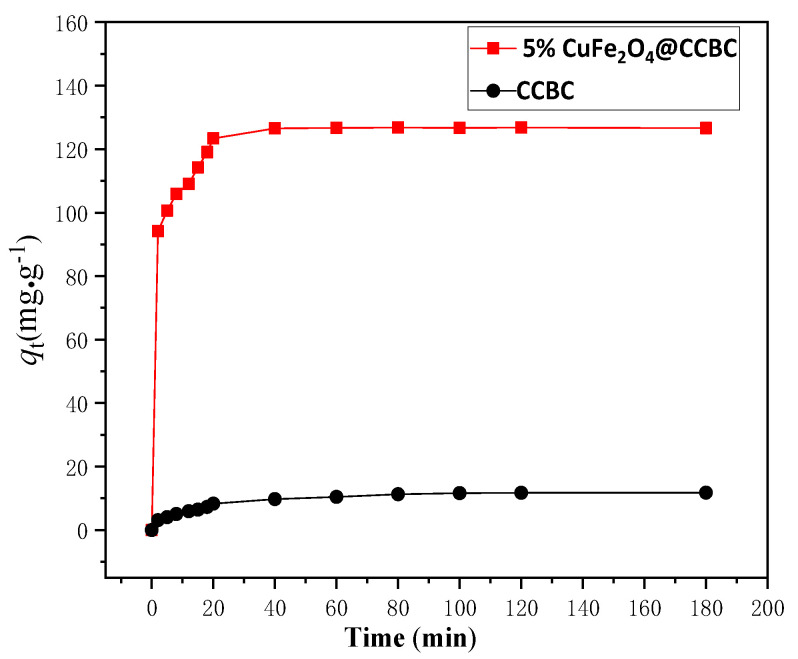
Process adsorption capacity over time.

Adsorption kinetic behavior is fundamental for selecting the optimal adsorbent material. For the typical kinetic adsorption test described in the Section 2.3, Figure 4 shows that *q*_t_ changed with the contact time. Obviously, Pb(II) adsorption on the tested materials rapidly improved at the initial phases, followed by a slow increase, and eventually reaching the equilibrium state at 40 min. Thus, this duration is considered as the equilibrium time period. The test data was fitted linearly according to the first-order and second-order models, respectively (Figure S5a,b). The estimated kinetic parameter values are presented in Table 1. The coefficient (R^2^) of the second-order model was higher than that of the first-order model, implying that it is reasonable to depict the adsorption kinetic behavior of CCBC and CuFe_2_O_4_@CCBC for Pb(II) based on the second-order kinetic model [25]. From the model parameters, the theoretical equilibrium adsorption capacity of CuFe_2_O_4_@CCBC(5%) (close to the measured value, as seen in Table 1) was 8.87 times higher than that of CCBC, while with a rate constant ratio advantage of 1.75. The results expressed that the adsorption kinetic performance of CCBC for the heavy metal pollutants could be significantly enhanced by the CuFe_2_O_4_ coating.

To elucidate the rate-determining steps toward Pb(II) adsorption on the adsorbents, the intraparticle diffusion model (Equation (12)) was used to estimate the experimental data. As shown in Figure S6, the plots are multi-linear, and a two-step adsorption process is observed, indicating that an initial and rapid sorption process first occurred on the surface, followed by a chemical adsorption process with functional groups and intraparticle diffusion of the lead ions. Obviously, none of the C constants approached zero (Table S2), suggesting that the chemical adsorption and intraparticle diffusion may not exclusively control Pb(II) adsorption [45].

### 2.4. Adsorption Thermodynamic Study

**Figure 5:**
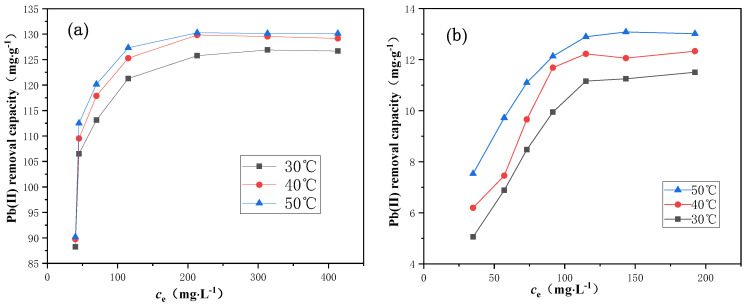
Adsorption isotherms for the Pb(II) adsorption on 5% CuFe_2_O_4_@CCBC (**a**) and CCBC (**b**).

It is vital to study the adsorption thermodynamic behavior for understanding the interaction of Pb(II) with the prepared materials in the aqueous solution [47]. Therefore, the equilibrium adsorption isotherms for the CuFe_2_O_4_@CCBC(5%) and CCBC adsorbents were conducted at 30, 40, and 50 °C, as shown in Figure 5a,b. Qualitative observation indicates that the equilibrium adsorption capacity increased first and subsequently reached a maximum value on increasing the residual Pb(II) concentration and temperature. The isothermal data were fitted using the Langmuir and Freundlich models for quantitative analysis, as displayed in Figure S6 and Table 2. From the correlation coefficients (R^2^), the Langmuir model was noted to be suitable to describe the adsorption equilibrium of Pb(II) on both CuFe_2_O_4_@CCBC and CCBC materials at the investigated temperatures. The R^2^ values for the Langmuir isotherms of the adsorption processes were observed to be greater than 0.95, especially for the Pb(II) adsorption on the CuFe_2_O_4_@CCBC(5%) sample, suggesting uniform monolayer adsorption. The results indicated that the *q*_m_ of CuFe_2_O_4_@CCBC(5%) was approximately 8 times higher than that of CCBC, which increased slightly with temperature, indicating the endothermic character of the adsorption process. Additionally, it has been reported that using partition coefficient (PC) as a metric for comparing the adsorption performance of adsorbents may be more objective and meaningful, mainly due to the ability to truly minimize the bias derivable from the use of the adsorption capacity concepts such as *q*_e_ and *q*_m_ [48,49,50,51]. Therefore, according to the paradigm established by Kim et al. [48], we estimated the PC values of both CuFe_2_O_4_@CCBC and CCBC materials accounting for initial Pb(II) concentration condition and Pb(II) adsorption capacities at 30 °C. Table S3 summarizes the obtained PC values. The PC values of CuFe_2_O_4_@CCBC(5%) and CCBC were found to decrease as the initial Pb(II) concentration increased and Pb(II) adsorption capacity increased. This trend was consistent with the phosphorus adsorption on the PEI-PEF and DC-PEI-PEF sorbents [48]. At a lower initial concentration (100 mg·L^−1^, 482.6 μM), the Pb(II) adsorption capacities of CuFe_2_O_4_@CCBC(5%) and CCBC were 88.25 and 9.95 mg·g^−1^, respectively. The *q*_e_ of CuFe_2_O_4_@CCBC(5%) was 8.9 times higher than that of CCBC. However, the calculated PC values for CuFe_2_O_4_@CCBC(5%) and CCBC were 0.444 and 0.022 mg·g^−1^∙μM^−1^, respectively. The PC of CuFe_2_O_4_@CCBC(5%) was noted to be 20.2 times to that of CCBC. Therefore, the observed significant difference in the PC values between CuFe_2_O_4_@CCBC(5%) and CCBC fully confirmed that the adsorption performance of CCBC for heavy metal ions like Pb(II) could be significantly improved by the CuFe_2_O_4_ coating. Moreover, it can be seen from Table S4 that CuFe_2_O_4_@CCBC(5%) exhibited superior adsorption capacity than other adsorbents reported in literature.

The standard thermodynamic change functions of the adsorption processes, including $\Delta G_{m}^{ο}$, $\Delta H_{m}^{ο}$, and $\Delta S_{m}^{ο}$, can be calculated from the Langmuir constant K_L_ and temperature by Equations (1)–(3), respectively [25,30,47]. From the obtained results presented in Table S3, $\Delta H_{m}^{ο}$ for the Pb(II) adsorption process on CuFe_2_O_4_@CCBC(5%) was 19.55 kJ·mol^−1^, while the value for the Pb(II) process on CCBC was 35.42 kJ·mol^−1^, indicating endothermic processes in both cases, with an enhanced temperature benefitting the adsorption process. A positive $\Delta G_{m}^{ο}$ value was obtained for the adsorption processes, indicating that the adsorption reaction could be non-spontaneous if the adsorbate, adsorbent, and resulting adsorption product were in their respective standard states. Furthermore, the positive $\Delta S_{m}^{ο}$ values indicated an increment in the degree of freedom of the adsorbed species, probably attributed to the hydroniums (H_3_O^+^) released into the bulk aqueous phase during the course of adsorption [30]. Based on the comprehensive analysis of the aforementioned adsorption standard thermodynamic functions, it is speculated that the adsorption reaction for removing Pb(II) by CuFe_2_O_4_@CCBC and CCBC is an entropy-driven process.

(1) $$\Delta G_{m}^{ο}=-RT\ln K_{L}$$

(2) $$\ln\frac{K_{L}(T_{2})}{K_{L}(T_{1})}=\frac{\Delta H_{m}^{ο}}{R}(\frac{1}{T_{1}}-\frac{1}{T_{2}})$$

(3) $$\Delta S_{m}^{ο}=\frac{\Delta H_{m}^{ο}-\Delta G_{m}^{ο}}{T}$$

### 2.5. Discussion on Mechanism of Pb(II) on CuFe_2_O_4_@CCBC

#### 2.5.1. Influence of Solution pH and Ion Strength

pH and ion strength can lead to the corresponding changes in the protonation/deprotonation of the surface groups of the adsorbent and speciation of the heavy metal species [16,25,46]. Figure S7 describes the adsorption results under different pH and NaCl concentrations. It is observed that the *q*_e_ values sharply increased as the pH was raised from 2.0 to 4.0, followed by a slight increment to the relative peak value (at pH = 6.0). On the other hand, further increase in the solution pH could have impacted the Pb(II) removal due to the formation of Pb(OH)_2_. In addition, a minor increase in the *q*_e_ was observed on increasing the ionic strength at different pH values. These trends imply that the adsorption process conforms to the mechanism of the inner surface complexation with accompanied ion exchange [52]. The FTIR analysis also indicated that CuFe_2_O_4_@CCBC contained effective hydroxyl (–OH) and carboxyl (–COOH) groups, thus, the cation exchange reactions could have occurred during the adsorption of Pb^2+^ ions, resulting in 1:1 or 1:2 surface complexes [25,52,53]. The surface adsorption reactions are speculated as reaction Equations (4)–(7), among which both reactions (5) and (7) might be more apt to occur, as the adsorption is an entropy-driven phenomenon.

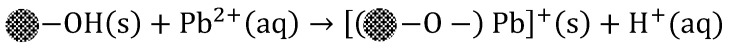
(4)

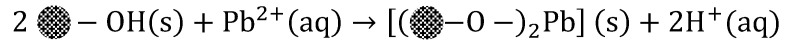
(5)

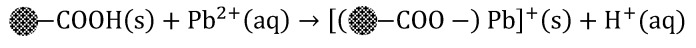
(6)

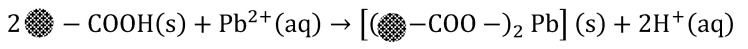
(7)

#### 2.5.2. XPS Analysis

**Figure 6:**
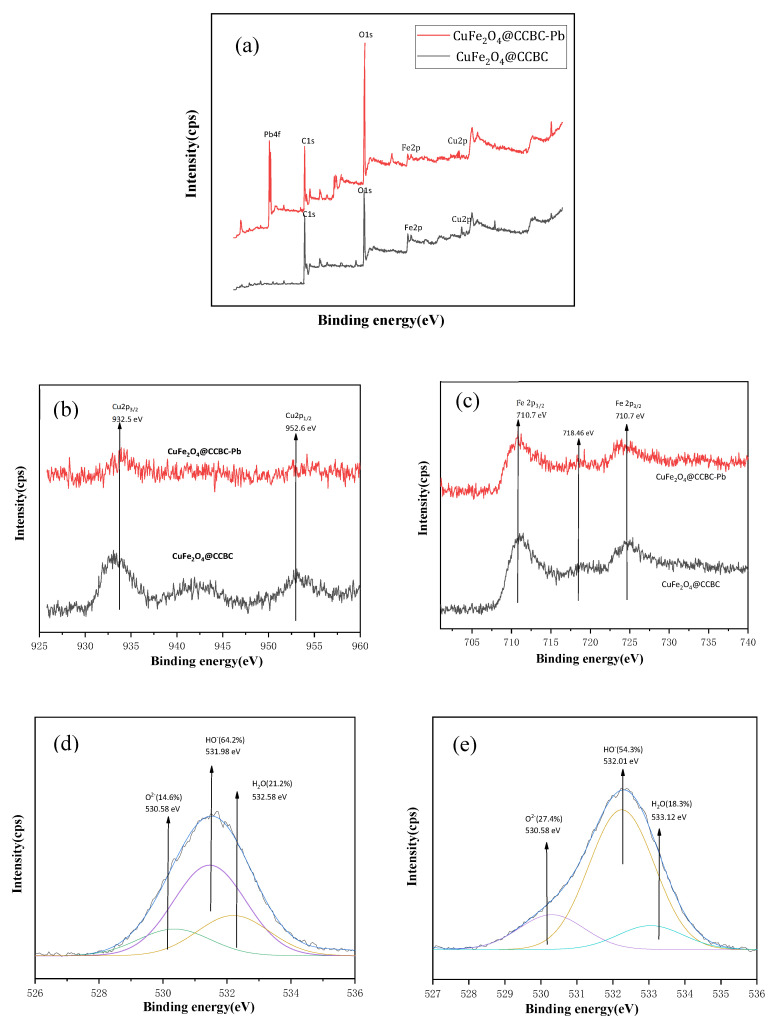
Comparison of XPS spectra of CuFe_2_O_4_@CCBC(5%) before and after the Pb(II) sorption: survey spectra (**a**), spectra of Cu2p core level region (**b**), spectra of Fe2p core level region (**c**), and spectra of O1s core level region (**d**,**e**).

A series of the XPS records are depicted in Figure 6, including the survey spectra (Figure 6a), Cu2p (Figure 6b) and Fe2p (Figure 6c) spectra of the core level regions as well as O1s region spectra (Figure 6d,e) before and after adsorption. Table 3 lists the main peak binding energy values, peak areas, and surface atomic compositions of each element, calculated from the XPS data. The F_A_ (named as area factor) values have also been presented in the table, which is defined as the coefficient of the main peak area of each element of the CuFe_2_O_4_@CCBC(5%)-Pb sample divided by that of CuFe_2_O_4_@CCBC(5%). The F_A_ values might be used to gain insights about the Pb(II) adsorption [25].

As presented in Figure 6a, two new peaks of Pb4f_7/2_ and Pb4f_5/2_ at 138.37 and 143.96 eV binding energy values were observed in the spectrum of the CuFe_2_O_4_@CCBC(5%)-Pb sample, which indicated that Pb(II) bonded to the sample surface. The presence of the Fe^3+^ and Cu^2+^ cations was identified by the spectra shown in Figure 6b,c, suggesting the face-centered cubic structure of the coated CuFe_2_O_4_ [44]. The Pb(II) adsorption resulted in a significant decrease in the height of the Cu2p and Fe2p peaks, especially Cu2p. This observation can be explained in detail based on the findings presented in Table 3. As can be seen from Table 3, seven elements (Cu, Fe, O, C, Si, Ca, and Na) were detected in the tested sample by XPS, while Pb was only found after the adsorption process. Among these elements, C had the highest content attributed to the biochar matrix, followed by the O element due to the abundant presence of the –OH and –COOH groups. On the other hand, Si, Ca, and Na are inherent impurities of the biochar, while both Cu and Fe are introduced by the coating. Obviously, the change in the atomic percentage of the elements is closely related to the Pb(II) sorption. The atomic ratio of each element could be reduced slightly, if the dilution effect caused by the Pb(II) adsorption was solely considered. In fact, a large reduction in the atomic ratio of Cu, Fe, C, and Na was observed; however, the atomic ratio of O and Si was increased, with the extent of Ca remaining almost unchanged. From Table 3, the order of the F_A_ values of the elements is as follows: F_A_(O) > F_A_(Si) > F_A_(Ca) > F_A_(C) > F_A_(Fe) > F_A_(Na) >> F_A_(Cu). It is speculated that a decrease in Na+ is caused by the ion exchange or dissolution during the Pb(II) sorption. The attenuation of Cu, Fe, and C can be ascribed to the bonding of their adjacent oxygen functional groups (–Cu–OH, –Fe–OH, –C–OH, or –COOH) with Pb^2+^ (as shown in the reaction Equations (11)–(14)), thus, the adsorbed Pb(II) significantly prevents the possibility of the incident electron colliding with the extranuclear electrons, resulting in the significant weakening of the XPS peaks. It is observed from Table 3 that the O atomic ratio increased from 25.47 to 28.78%, and the F_A_ value was the highest, which can be explained by the fact that the Pb^2+^, Cu^2+^, and Fe^3+^ ions are coordinated by the O^2−^ ions, as more O is exposed to the surface of the particles. The XPS spectra of the O1s regions of the CuFe_2_O_4_@CCBC(5%) and CuFe_2_O_4_@CCBC(5%)-Pb samples are presented in Figure 6d,e, which can be fitted into three contributions [54,55], indicating that the proportion of the lattice oxygen (O^2−^) increased (from 14.6 to 27.4%); however, the content of the surface hydroxyl groups (OH^−^) and adsorbed water (H_2_O) was reduced (from 64.2 and 21.2% to 54.3 and 18.3%, respectively). The results further demonstrate that the oxygen-bonding functional groups, such as –OH and –COOH, are the main adsorption sites towards Pb(II). As for the increased surface Si, it probably resulted from the dissolution of the impurities containing the quartz particles.

#### 2.5.3. FTIR Characterization

The FTIR spectra of CuFe_2_O_4_@CCBC(5%) and CuFe_2_O_4_@CCBC(5%)-Pb are presented in Figure S8, in order to observe the changes in the characteristic peaks of the functional groups (potential adsorption sites for Pb(II)). A significant decrease in the intensity of the bands corresponding to the hydroxyl groups (about 3435, 870, 970, and 1457 cm^−1^) was observed, and the two absorbance peaks of the carboxyl groups at 1640 and 1560 cm^−1^ almost disappeared due to the lead ions adsorption. The results demonstrate that the lead ions are chemically adsorbed with the hydroxyl and carboxyl groups in the CuFe_2_O_4_@CCBC structure, which is consistent with the mechanism proposed above.

## 3. Materials and Methods

### 3.1. Synthesis of CuFe_2_O_4_@CCBC Composites

The raw corncob biochar was provided by Hebei Batu Biotechnology Co., Ltd. (Xingtai, China). The sample was washed and used as a blank sample (denoted as CCBC). Further, it was used as a precursor to prepare the magnetic biochar composites. The magnetic CuFe_2_O_4_-loaded corncob biochar (denoted as CuFe_2_O_4_@CCBC) was prepared by combining the two-step impregnation of CCBC with the pyrolysis of oxalate [25]. Three CuFe_2_O_4_@CCBC composites with different mass contents of CuFe_2_O_4_ (3, 5, and 8%) were achieved according to the following steps: Fe(NO_3_)_3_·9H_2_O and Cu(NO_3_)_2_·3H_2_O at Cu:Fe molar ratios of 1:2 were dissolved in 7 mL of 80% ethanol solution. Next, 7 mL of the solution was added dropwise to 10 g of CCBC. The mixture was uniformly mixed and allowed to stand for 30 min before placing in an air-drying oven at 65 °C for 12 h. 7.2 mL of the saturated oxalic acid (C_2_H_2_O_4_·2H_2_O, A.R.) solution was added dropwise to the dried solid. Similar to the earlier procedure, the mixture was uniformly mixed and allowed to stand for 30 min before placing in an air-drying oven at 65 °C for 12 h. Subsequently, the mixture was tiled in a quartz boat and calcined at 300 °C for 1 h in a horizontal tube furnace. The calcined samples were sieved and placed in a desiccator for further characterization.

### 3.2. Characterization of CuFe_2_O_4_@CCBC and CCBC

The crystallographic structure of the samples was identified from the X-ray diffraction (XRD) patterns recorded in the 2θ range of 10–80° using D8 ADVANCE diffractometer (Bruker AXS, Karlsruhe, Germany). Fourier transform infrared (FTIR) spectra of the adsorbents were acquired using KBr pellets in the wavelength region of 4000–400 cm^−1^ via FTIR spectrometer (Nicolet 6700, Theromo Fisher, Waltham, MA, America). The absorbent textural parameters were obtained using the Brunauer-Emmett-and-Teller (BET) multipoint approach by employing the surface area and porosity analyzer (Kubo-X1000, Beijing Electronic Technology Co., Ltd., Beijing, China). The material morphology was observed on a scanning electron microscope (SEM; S-4800, Hitachi Ltd., Tokyo, Japan), while elemental composition of the material surface was measured using its accompanying INCA Energy 350 spectrometer. Photoelectron spectroscopy (Escalab 250Xi, ThermoFisher, Waltham, MA, American) was used to investigate the chemical composition and state of the material surface. In addition, the magnetic parameters of the biochar composite were measured using a vibrating sampling magnetometer (VSM; MPMS-3, San Diego, CA, America).

### 3.3. pH Drift Experiment

The pH of the zero-point charges (pH_ZPC_) of the adsorbents was determined using the following procedure [45]: (1) 25 mL of 0.01 mol·L^−1^ NaCl solution was poured in a 50 mL plastic centrifuge tube. The initial solution pH (pH_0_) was adjusted to discrete initial values between 2.0 and 11.0, and 0.05 g of the sample was subsequently added to the tube; (2) The plastic tube was filled with N_2_ to minimize the effect of CO_2_ on pH, followed by shaking for 20 h at 40 °C; (3) The final solution pH was immediately measured. The difference between the final pH and the pH_0_, named as ΔpH, was plotted against pH_0_. The solution pH at which the curve crosses the line of ΔpH = 0 was perceived as the pH_ZPC_ of the samples.

### 3.4. Batch Adsorption Experiments

The Pb(II) stock sample with a concentration of 1000 mg·L^−1^ was acquired by dissolving Pb(NO_3_)_2_ in the measured volume of DI water, which was further attenuated to the required concentrations (80–500 mg·L^−1^). A series of 30 mL Pb(II) solutions were added to 50 mL plastic centrifuge tubes, with adjustment of the initial pH to the specified value (the pH was 5.0 for most of the studied systems, while the values of 2.0, 3.0, 4.0, 5.0, and 6.0 were attained to study the pH effect). 0.01 mol·L^−1^ HNO_3_ or NaOH solution was used to regulate the initial pH of the solutions. Afterwards, the batch experiments were conducted by introducing 20 mg of the adsorbent sample to the pH-preadjusted solution. The adsorption tubes were swiftly placed in a shaking incubator and continuously shaken at 30 °C for 8 h to achieve equilibrium. About 5 mL solution was sampled from the tubes and filtered using 0.45 μm membrane syringe filter. The remaining Pb(II) concentration in the aqueous filtrate was measured using a flame atomic absorption spectrophotometer (T6, Beijing Pu Analysis General Instrument Co., Ltd., Beijing, China) so as to assess the Pb(II) equilibrium adsorption capacity (*q*_e_). The influence of the CuFe_2_O_4_ loading, initial pH and ionic strength on the Pb(II) adsorption was analyzed according to the above mentioned procedure. Additionally, the kinetic experiments were performed at an initial concentration of 500 mg·L^−1^ for different contact time intervals (2, 5, 8, 12, 15, 18, 20, 40, 60, 80, 120, and 180 min). The residual Pb(II) concentration of the samples was determined to estimate the Pb(II) process adsorption capacity (*q*_t_). Here, Equations (8) and (9) were applied to calculate *q*_t_ (mg·g^−1^) and *q*_e_ (mg·g^−1^).

(8) $$q_{t}=\frac{c_{0}-c_{t}}{m}$$

(9) $$q_{e}=\frac{\left(c_{0}-c_{t}\right)\times V}{m}$$

where c_0_, c*_t_* and c_e_ are the initial, t-time residual and equilibrium concentration of Pb(II) (mg·L^−1^). *V* is the solution volume (mL) and *m* is the adsorbent mass (mg).

The kinetic experimental data were treated using the first-order (Equation (10)), second-order (Equation (11)) and intraparticle diffusion models (Equation (12)) [56], respectively. Furthermore, typical isothermal experiments were performed using a group of Pb(II) solutions with different initial concentration at 30, 40, and 50 °C respectively. The obtained data were fitted to the linear Langmuir and Freundlich equations, defined as Equations (13) and (14), respectively.

(10) $$\log\left(q_{e}-q_{t}\right)=\log q_{e}-0.434k_{1}t$$

(11) $$\frac{t}{q_{t}}=\frac{1}{k_{2}q_{e}^{2}}+\frac{t}{q_{e}}$$

(12) $$q_{t}=k_{i}\sqrt{t}+C$$

(13) $$\frac{c_{e}}{q_{e}}=\frac{1}{q_{m}K_{L}}+\frac{c_{e}}{q_{m}}$$

(14) $$\ln q_{e}=\ln K_{F}+\frac{1}{n}\ln c_{e}$$

where the parameters k_1_ (min^−1^), k_2_ (g·mg^−1^·min^−1^) and k*_i_* (mg·g^−1^·min^−1/2^) represent the rate constants of the first-order, second-order, and intra-particle diffusion models, respectively. C refers to the boundary layer thickness for the intra-particle diffusion model. The parameter *q*_m_ represents the maximum Pb(II) adsorption amount and K_L_ is Langmuir constant. K_F_ and n are Freundlich empirical constants.

### 3.5. Partition Coefficient Estimation

With the aforementioned batch isotherm experimental data, the following Equation (15) was employed to calculate the partition coefficients (PC).

(15) $$\mathrm{PC}=\frac{q_{e}}{c_{e}}$$

where the *q*_e_ and *c*_e_ are the Pb(II) adsorption capacity (mg·g^−1^) of the adsorbents and Pb(II) concentration (μmol·L^−1^, μM) in the solution at adsorption equilibrium, respectively.

## 4. Conclusions

Magnetic CuFe_2_O_4_-loaded corncob biochar (CuFe_2_O_4_@CCBC) was prepared by combining the two-step impregnation of CCBC with the pyrolysis of oxalate. CuFe_2_O_4_, with a face-centered cubic crystal phase, was homogeneously coated on the surface of CCBC. The CuFe_2_O_4_@CCBC(5%) sample exhibited a specific area of 74.98 m^2^·g^−1^, saturation magnetization of 5.75 emu·g^−1^ and pH_ZPC_ of 7.0. CuFe_2_O_4_@CCBC with 5% loading amount was observed to be the most effective material for the removal of Pb(II) from wastewater. The pseudo-second kinetic and Langmuir models suitably fitted the Pb(II) adsorption by CuFe_2_O_4_@CCBC. The rate constant and maximum adsorption capacity of Pb(II) by CuFe_2_O_4_@CCBC(5%) at 30 °C were observed to be 7.68 × 10^−3^ g·mg^−1^·min^−1^ and 132.10 mg·g^−1^, respectively, which were significantly higher than CCBC (4.38 × 10^−3^ g·mg^−1^·min^−1^ and 15.66 mg·g^−1^). The adsorption reaction for removing Pb(II) by CuFe_2_O_4_@CCBC was an endothermic entropy-driven process. The multiple analytical characterizations illustrated that the specific area and extent of oxygen-bonding groups (M–OH and –COOH) increased after generating the CuFe_2_O_4_ coating, thus, resulting in enhanced Pb(II) adsorption ability. The main adsorption mechanism could, thus, be the conjunction of the ion exchange and inner surface complexation. The study suggests that the magnetic CuFe_2_O_4_-loaded biochar can be an efficient and eco-friendly adsorbent for the heavy metal abatement.

## Figures and Tables

**Table 1:** Adsorption kinetic fitting results.

		First-order			Second-order		
	*q*_e,exp_ (mg·g^−1^)	*q*_e_ (mg·g^−1^)	k_1_ (min^−1^)	*R* ^2^	*q*_e_ (mg·g^−1^)	k_2_ (g·mg^−1^·min^−1^)	*R* ^2^
CCBC	11.50	11.33	1.42 × 10^−2^	0.9611	14.10	4.38 × 10^−3^	0.9842
CuFe_2_O_4_@CCBC(5%)	126.67	120.24	6.60 × 10^−2^	0.8998	125.13	7.68 × 10^−3^	0.9991

*q*_e,exp_: experimental equilibrium adsorption capacity, *q*_e_: theoretical equilibrium adsorption capacity, k_1_: first-order rate constant, k_2_: second-order rate constant, *R:* correlation coefficient

**Table 2:** Adsorption isotherm parameters and correlation coefficients of the Pb(II) adsorption.

	Langmuir Constants				Freundlich Constants		
	*T* (K)	*q*_m_ (mg·g^−1^)	K_L_ (L·mg^−1^)	R^2^	K_F_ (mg·g^−1^)(L·mg^−1^)^1/n^	n	R^2^
**CCBC**	303	15.66	0.013	0.9891	1.26	0.087	0.8835
	313	16.66	0.018	0.9544	1.80	0.021	0.8207
	323	16.93	0.032	0.9533	3.03	0.016	0.8576
**CuFe_2_O_4_@CCBC(5%)**	303	132.10	0.059	0.9997	63.89	8.21	0.7339
	313	134.23	0.078	0.9995	66.26	8.34	0.6851
	323	134.41	0.096	0.9989	68.80	8.74	0.6315

*q*_m_: maximum adsorption amount, K_L_: Langmuir constant, K_F_ and n: Freundlich empirical constants.

**Table 3:** XPS parameters of CuFe_2_O_4_@CCBC(5%) before and after the Pb(II) sorption.

		Cu2p	Fe2p	O1s	C1s	Si2p	Ca2p	Na1s	Pb4f
CuFe_2_O_4_ @CCBC(5%)	BE (eV)	933.24	710.91	530.80	284.13	101.99	346.98	1070.39	-
	Peak area (CPS·eV)	111,990	284,117	494,988	452,400	31,376	82,267	66,321	-
	Atomic%	1.43	3.02	25.47	62.13	3.67	2.40	1.88	-
CuFe_2_O_4_ @CCBC(5%)-Pb	BE (eV)	933.62	710.34	531.23	284.09	102.22	347.41	1070.80	138.37
	Peak area (CPS·eV)	10,245	229,330	762,573	389,258	44,052	85,689	51,115	69,3037
	Atomic(%)	0.54	2.36	28.78	56.32	4.07	2.37	1.61	3.95
F_A_		0.09	0.81	1.54	0.86	1.40	1.04	0.77	∞

BE: binding energy, F_A_: area factor.

## Supplementary Materials

The following are available online, Figure S1: SEM images of CCBC (a) and CuFe_2_O_4_@CCBC (5%) (b) as well as mappings of Cu (c) and Fe (d); Figure S2: XRD patterns of CuFe_2_O_4_@CCBC(5%) and CCBC; Figure S3: Nitrogen adsorption/desorption isotherms (a) and pore-size distribution diagram (b) of the samples; Figure S4: pH drift curves of the samples; Figure S5: (a) Adsorption kinetics fitted with the pseudo-first-order model of Pb(II), (b) Adsorption kinetics fitted with the pseudo-second-order model of Pb(II), (c) Adsorption kinetics fitted with the intra-particle diffusion kinetics model of Pb(II); Figure S6: (a) Langmuir and (c) Freundlich isotherm models fitted on the Pb(II) adsorption for CuFe_2_O_4_@CCBC(5%); (b) Langmuir and (d) Freundlich isotherm models fitted on the Pb(II) adsorption for CCBC. (Contact time = 24 h, pH = 5.0.); Figure S7: Influence of pH and ion strength on Pb(II) sorption; Figure S8: FTIR spectra of CuFe_2_O_4_@CCBC(5%) and CuFe_2_O_4_@CCBC(5%)-Pb; Table S1. Texture properties of different samples; Table S2: Comparison of the adsorption performance of typical adsorbents for Pb(II); Table S3: Thermodynamic parameters for the Pb(II) adsorption by CuFe_2_O_4_@CCBC and CCBC.
