# Supplementary material for: Study on the Adsorption of CuFe2O4-Loaded Corncob Biochar for Pb(II)
Source: Molecules. 2020 Jul 29;25(15):3456. doi: 10.3390/molecules25153456 (PMC7435881; doi:10.3390/molecules25153456)
Supplement: Supplementary file 1 [file molecules-25-03456-s001.pdf]

molecules

MDPI

Supplementary Material

Study on the adsorption of $CuFe_2O_4$-loaded corncob biochar for Pb(II)

Tianci Zhao[1], Xiaolong Ma[2], Hao Cai [1], Zichuan Ma[1,*] and Huifeng Liang[3 *]

[1] College of Chemistry and Material Science, Hebei Normal University, Shijiazhuang 050024, Hebei, PR China; 1019909378@qq.com (T. Z.); hao_cai_515@163.com (H. C.); mazc@hebtu.edu.cn (Z. M.) ;

[2] School of Environmental Science and Engineering, Hebei University of Science and Technology, Shijiazhuang 050018, Hebei, PR China; maxiaolong2410@163.com (X. M.) ;

[3] College of Chemistry and Chemical Engineering, Xingtai University, Xingtai 054001, Hebei, PR China; lianghuifeng6612@163.com (H. L.) ;

* Correspondence: mazc@hebtu.edu.cn ; lianghuifeng6612@163.com ;Tel.: +86 311 80787400

Table S1. Texture properties of different samples[a].

Samples	Specific surface area ($m^2·g^{-1}$)	Pore volume ($cm^3·g^{-1}$)	Pore size (nm)
**CCBC**	17.1	0.039	7.93
**$CuFe_2O_4$@CCBC(3%)**	30.7	0.067	2.57
**$CuFe_2O_4$@CCBC(5%)**	75.0	0.082	2.44
**$CuFe_2O_4$@CCBC(8%)**	60.7	0.057	1.89

[a] Pore volume was measured at 0.99 of p/p_0, pore size was calculated from the absorptive branch by BJH method.

Table S2. Kinetics parameters calculated from intra-particle diffusion model for Pb(II) adsorption onto samples (k_{i1}: $mg·g^{-1}·min^{-1/2}$, k_{i2}: $mg·g^{-1}·min^{-1/2}$).

samples	k_{i1} ($mg·g^{-1}·min^{-1/2}$)	C_1	R^2	k_{i2} ($mg·g^{-1}·min^{-1/2}$)	C_2	R^2
**CCBC**	1.39	1.09	0.9959	0.83	4.22	0.9166
**$CuFe_2O_4$@CCBC(5%)**	7.61	83.59	0.9930	1.84	113.44	0.7159

Table S3. Partition coefficient (PC) for Pb(II) adsorption onto CCBC and $CuFe_2O_4$@CCBC(5%).

amples	Sorbent density ($g·L^{-1}$)	Initial Pb(II) Concentration (μM)	Final Pb(II) Concentration (μM)	Removal rate (%)	Equilibrium Pb(II) sorption capacity ($mg·g^{-1}$)	Partition coefficient ($mg·g^{-1}·μM^{-1}$)
**CCBC**	0.67	193.1	176.8	8.4	5.06	0.029
		289.6	267.4	7.6	6.89	0.026
		386.1	358.8	7.1	8.48	0.024
		482.6	450.6	6.6	9.95	0.022
		579.2	543.2	6.2	11.16	0.020
		723.9	687.7	5.0	11.25	0.016
		965.2	926.9	4.0	11.50	0.012
**$CuFe_2O_4$@CCBC(5%)**	0.67	482.6	198.7	58.8	88.25	0.444
		579.2	239.4	58.7	106.05	0.443
		723.9	359.9	50.3	113.15	0.314
		965.2	575.0	40.4	121.29	0.211
		1447.9	1043.2	28.0	125.78	0.120
		1930.5	1522.2	21.2	126.90	0.083
		2413.1	2005.6	16.9	126.98	0.063

Table S4. Comparison of the adsorption performance of typical adsorbents for Pb(II).

Adsorbent sample	Adsorption capacities ($mg\cdot g^{-1}$)	Reference
**MnFeOx@CCBC**	99.60	[1]
**Magnetic Douglas fir biochar (MBC)**	26.00	[2]
**Cannabis biochar (CA400)**	106.39	[3]
**A H3PO4-modified biochar (CFCP)**	55.42	[4]
**anaerobic digestion sludge biochar (ADSBC600)**	51.77	[5]
**CuFe2O4@CCBC(5%)**	132.10	This study

Table S5. Thermodynamic parameters for the Pb(II) adsorption by $CuFe_2O_4$@CCBC and CCBC.

	**$CuFe_2O_4$@CCBC(5%)**			**CCBC**		
	ΔG_m^o **(kJ·mol-1)**	ΔH_m^o **(kJ·mol-1)**	ΔS_m^o **(J·mol-1·K-1)**	ΔG_m^o **(kJ·mol-1)**	ΔH_m^o **(kJ·mol-1)**	ΔS_m^o **(J·mol-1·K-1)**
**303 K**	7.13		41.02	10.89		80.80
**313 K**	6.65	19.55	41.23	10.39	35.42	79.99
**323 K**	6.30		41.03	9.29		80.84

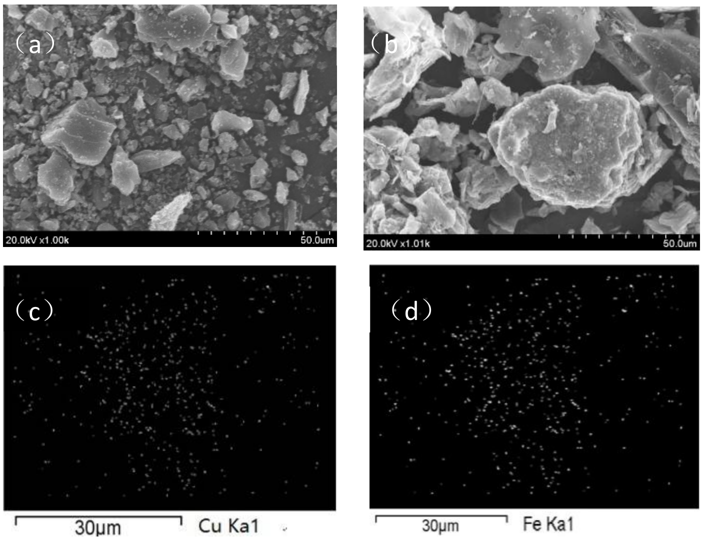

Figure S1. SEM photos of CCBC (a) , $CuFe_2O_4$@CCBC (5%) (b) and mappings of Cu (c), Fe (d).

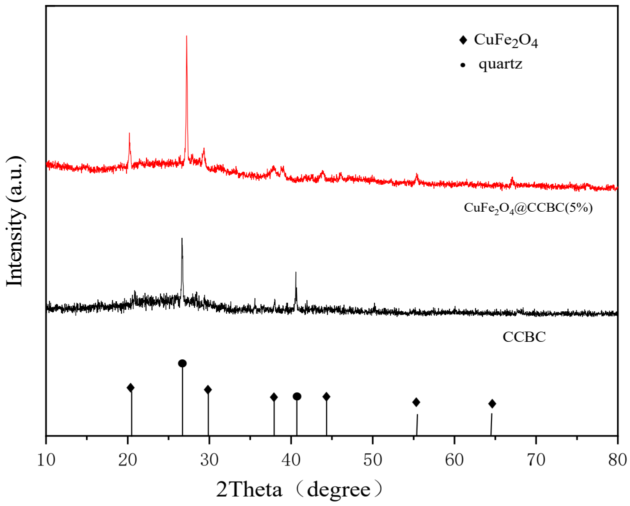

Figure S2. XRD patterns of $CuFe_2O_4$@CCBC (5%) and CCBC.

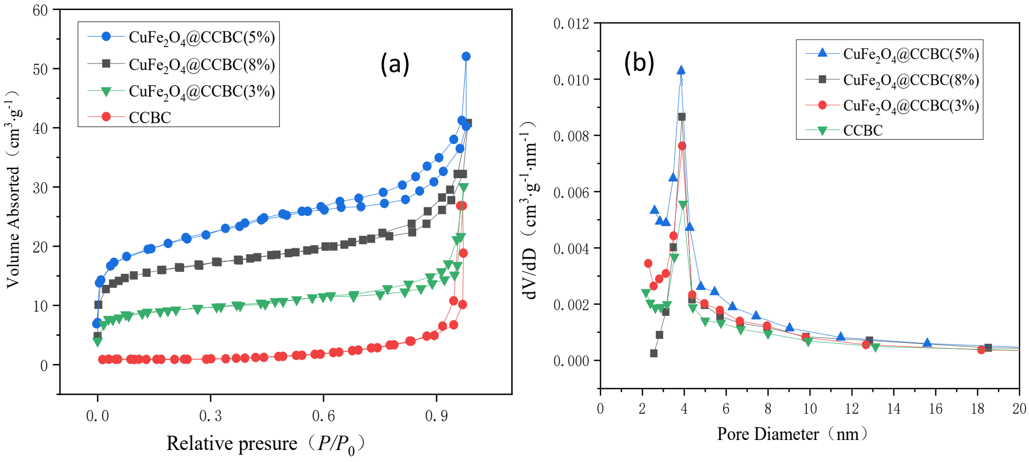

Figure S3. Nitrogen adsorption/desorption isotherms (a) and pore-size distribution diagram (b) of the samples.

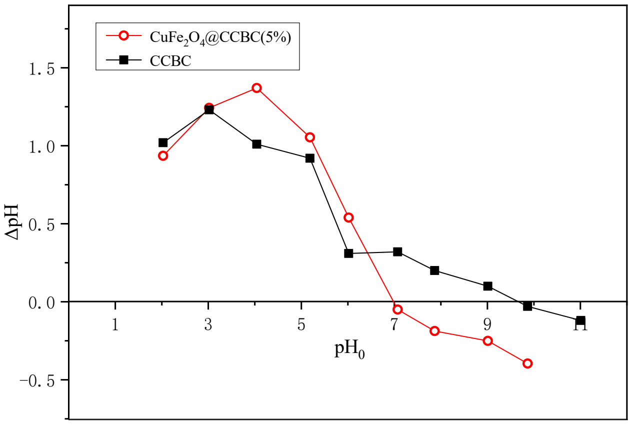

Figure S4. pH drift curves of the samples.

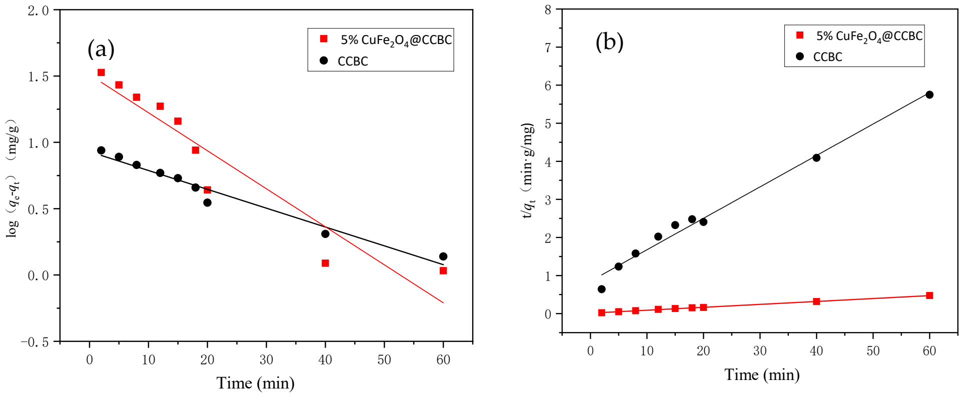

Figure S5. (a) Adsorption kinetics fitted with the pseudo-first-order model of Pb(II), (b) Adsorption kinetics fitted with the pseudo-second-order model of Pb(II). Initial Pb(II) concentration of 500 g·L^{-1} and 30±1 ℃.

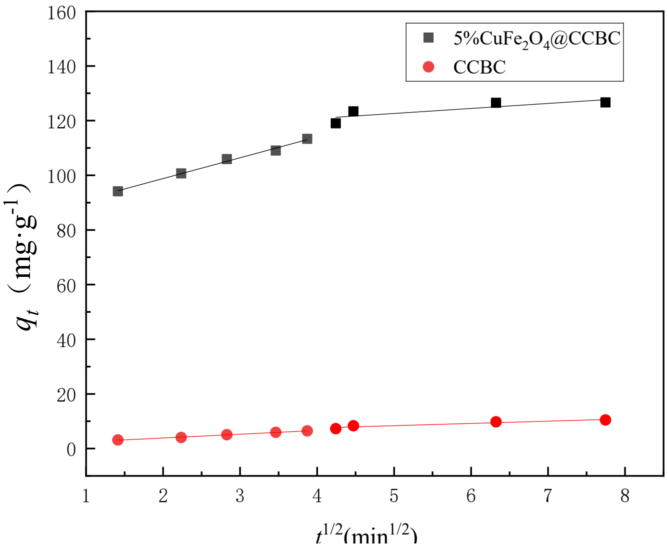

Figure S6. Intraparticle diffusion plot of the Pb(II) adsorption. Initial Pb(II) concentration of 500 g·L^{-1} and 30±1 ℃.

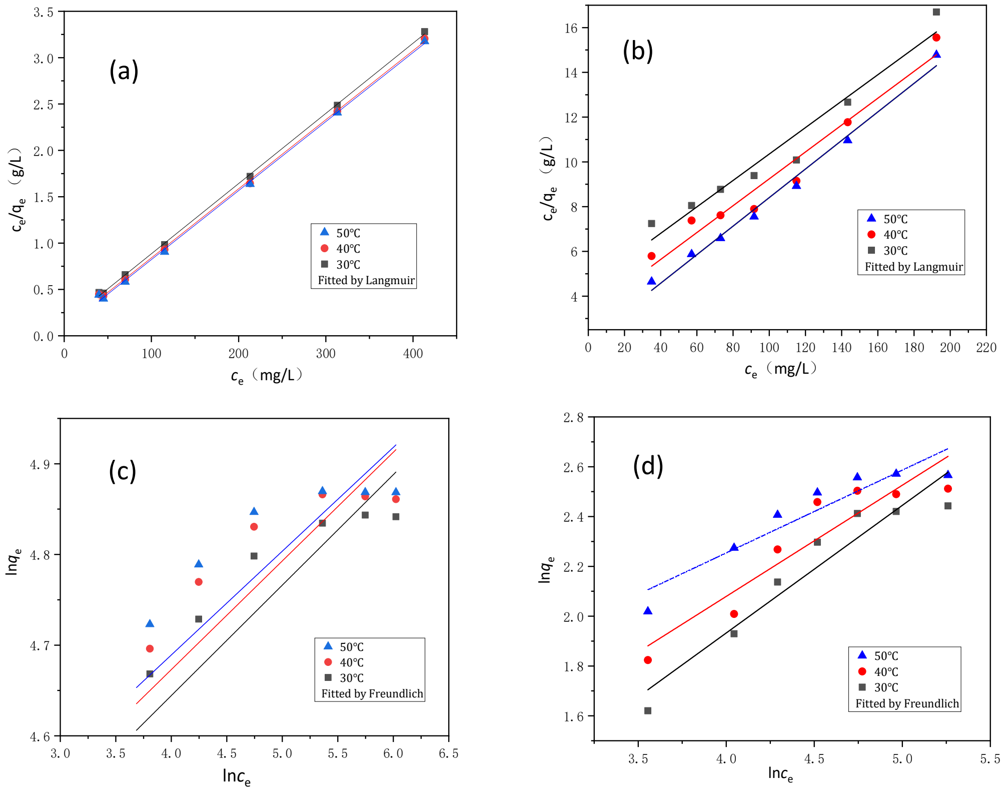

Figure S7. (a) Langmuir and (c) Freundlich isotherm models fitted onto the Pb(II) adsorption for $CuFe_2O_4$@CCBC(5%); (b) Langmuir and (d) Freundlich isotherm models fitted onto the Pb(II) adsorption for CCBC. (Contact time = 24 h, pH = 5.0.).

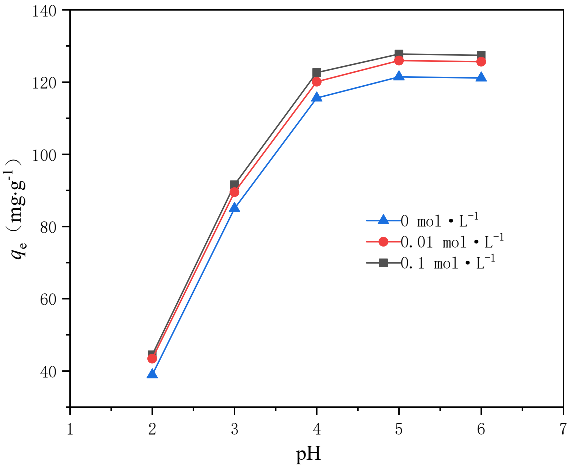

Figure S8. Influence of pH and ion strength on Pb(II) sorption.

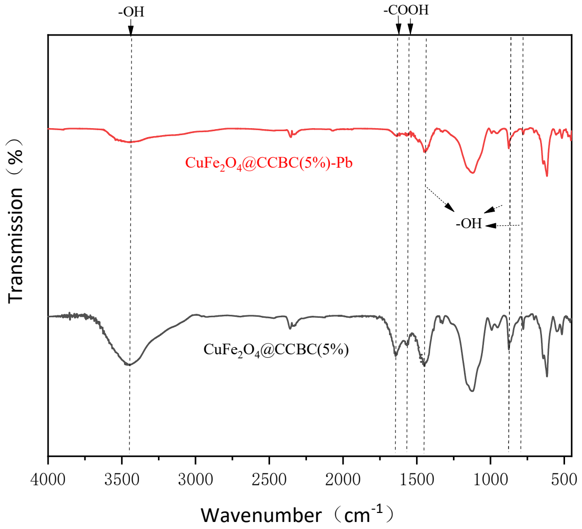

Figure S9. FTIR Spectra of the $CuFe_2O_4$@CCBC(5%) and $CuFe_2O_4$@CCBC(5%)-Pb.

Supplementary Materials References

1. Zhao, T.C.; Zhou, S.Z.; Ma, X.L.; Ma, Z.C. Study on the adsorption of Pb^{2+} by $MnFeO_x$ -loaded corncob biochar. *Acta Scientiae Circumstantiae.* 2019, 39,2997-3009.
2. Karunanayake, A. G.; Todd, O. A.; Crowley, M.; Ricchetti, L.; Pittman, C. U.; Anderson, R.; Mohan, D.; Mlsna, T., Lead and cadmium remediation using magnetized and nonmagnetized biochar from Douglas fir. *Biochem. Eng. J.* 2018, 331, 480-491.
3. Omidi, A. H.; Cheraghi, M.; Lorestani, B.; Sobhanardakani, S.; Jafari, A., Biochar obtained from cinnamon and cannabis as effective adsorbents for removal of lead ions from water. *Environ. Sci. Pollut. Res.* 2019, 26, (27), 27905-27914.
4. Chen, H.; Li, W.; Wang, J.; Xu, H.; Liu, Y.; Zhang, Z.; Li, Y.; Zhang, Y., Adsorption of cadmium and lead ions by phosphoric acid-modified biochar generated from chicken feather: Selective adsorption and influence of dissolved organic matter. *Bioresour. Technol.* 2019, 292, 121948.
5. Ho, W. C. J.; Tay, Q.; Qi, H.; Huang, Z.; Li, J.; Chen, Z., Photocatalytic and Adsorption Performances of Faceted Cuprous Oxide (Cu(2)O) Particles for the Removal of Methyl Orange (MO) from Aqueous Media. *Molecules*. 2017, 22, 677.
